# Application of Psychoeducation-Based Model Group Work in Continuous Treatment of Patients With Bipolar Disorder in Remission

**DOI:** 10.31083/AP43989

**Published:** 2025-06-18

**Authors:** Xuemin Shi, Suilin Jia, Lingkai Yang, Zhipeng Yin, Bowen Yin, Guangdong Chen

**Affiliations:** ^1^Department of Behavior Medicine of Wenzhou Seventh People’s Hospital, 325000 Wenzhou, Zhejiang, China; ^2^Department of Children Psychiatry of Wenzhou Seventh People’s Hospital, 325000 Wenzhou, Zhejiang, China; ^3^Department of General Psychiatry of Wenzhou Seventh People’s Hospital of Wenzhou, 325000 Wenzhou, Zhejiang, China; ^4^Mental Comprehensive First Ward of Wenzhou Seventh People’s Hospital of Wenzhou, 325000 Wenzhou, Zhejiang, China

**Keywords:** psychoeducation, bipolar disorders, patient compliance, awareness

## Abstract

**Objective:**

To explore the value of psychoeducation-based group work in the continuous treatment of patients with bipolar disorder in remission.

**Methods:**

From December 2020 to March 2022, 60 outpatients with remission-stage bipolar disorder were enrolled in the trial. All enrolled subjects were randomly and single-blindly divided into a study group and a control group at a 1:1 ratio. The control group was treated with general drug therapy, while the study group was treated with group psychological education combined with drug therapy. To analyze the treatment adherence of patients in the two groups, and to compare the changes in self-awareness and attitude toward treatment questionnaire (ITAQ) scale scores, Morisky medication adherence scale scores, Self-rating Depression Scale (SDS) total scores, and subscale scores before and after the intervention in the two groups.

**Results:**

The mean score for treatment compliance in the study group was 4.2 ± 0.3, which was significantly higher than that in the control group (4.2 ± 0.3 *vs*. 3.2 ± 0.5, *p* < 0.001). The ITAQ score in the study group following the intervention was significantly higher than that in the control group (18.5 ± 3.2 *vs*. 12.7 ± 2.7, *p* < 0.001), as well as the study group prior to the intervention (*p* < 0.001). Morisky scale scores after intervention were significantly higher than those in the control group (6.9 ± 1.0 *vs*. 5.5 ± 0.8, *p* < 0.001) and the study group before intervention (*p* < 0.001). Sheehan Disability Scale 1 (SDS1), SDS2, SDS3, and SDS scores after intervention were 8.5 ± 1.2, 8.0 ± 1.5, 7.9 ± 2.0, and 25.5 ± 4.3, respectively, all of which were significantly higher than those in the control group (all *p* < 0.001). The proportion of positive coping style for bipolar disorder in the study group was significantly higher than that in the control group (93.3% *vs*. 50.0%, *p* = 0.001).

**Conclusion:**

Continuous intervention using psychoeducation-based model group work in patients with bipolar disorder in the remission stage can significantly improve treatment compliance, improve insight and treatment attitudes, ensure compliance with drug therapy, and reduce the degree of mental disability.

## Main Points

1. This study explores the application value of the psychoeducation-based group work model in the continued treatment of patients with bipolar disorder in remission.

2. The study found that the psychoeducation-based group work study group had significantly better treatment compliance, self-awareness and treatment attitudes, and medication compliance than the control group.

3. The psychoeducation-based group work study group also scored significantly better than the control group in the Sheehan Disability Scale, indicating that this model can effectively improve the overall treatment of patients.

4. Patients in the study group showed more positive coping methods, indicating that group psychoeducation helps change the patient’s subjective attitude towards the disease.

5. This study has laid a preliminary foundation for the application of group psychoeducation in the continued treatment of bipolar disorder and provides a basis for further optimization and improvement of this model in the future.

## 1. Introduction

Bipolar disorder (BD) is a serious mood disorder alternating between depression and mania. Its recurrence rate after treatment is extremely high, ranking in the top six disability rates of physical and mental diseases, and its mortality rate is steadily the first in the mortality rate of mental disorders [1]. Bipolar disorder is considered to be a biological disorder with drug intervention as the main treatment [2], but there are many drug complications clinical application, and treatment compliance is low, which seriously affects the clinical effect [3].

Psychological intervention is a non-pharmacological treatment and plays an increasingly important role in the treatment of bipolar disorder. Psychotherapy can help patients improve their self-knowledge, emotional regulation, and coping ability, and help to reduce the risk of relapse. Social support can help patients obtain emotional support, information, and practical help to improve the quality of life [4].

However, traditional psychotherapy tends to be individual therapy, lacking teamwork and interaction, which makes it difficult to meet the diverse needs of BD patients [5]. Therefore, exploring a group work model, based on group psychoeducation, for the continuation of treatment for BD patients is of great clinical significance. Group psychoeducation improves patients’ knowledge of the disease and their ability to manage the disease by teaching them and their families disease information and coping skills, thereby reducing relapse and improving quality of life. Group psychoeducation mainly includes cognitive-behavioral therapy, interpersonal therapy, family therapy, and other forms. These modes of intervention emphasize patient-centeredness and enhance patients’ self-awareness and coping ability through interaction and sharing. Compared with individual psychological interventions, group psychoeducation has the advantages of strong mutual support, resource saving, and improved compliance, etc. However, there is no empirical evidence showing that group psychoeducation can help patients to improve their self-awareness and coping ability [6].

In view of the large clinical workload of psychiatrists, as well as their energy and professional limitations [7], it is not possible to provide more effective comprehensive psychosocial treatment for patients with bipolar disorder in addition to pharmacological intervention. At this point, the professional social worker applies theory to practice, through professional practical support [8], to better provide professional services to patients with mental illness, provide psychological education on the disease, explore its intrinsic potential, and establish patients’ confidence in social reintegration, helping them to better integrate into society and have professional advantages [9]. Therefore, it is particularly important for social workers to enter psychiatric hospitals to provide professional rehabilitation services for patients with mental illness [10].

The author of this article started to carry out social services in the clinic in 2015, accumulated a lot of project service experience in the early stages, and also gained a comprehensive and profound understanding of patients with mental illness. Therefore, this study provides psychoeducational group service intervention for patients with bipolar disorder in remission and conducted a cohort-control study with conventional drug therapy to examine the use of group-based psychoeducation in patients with bipolar disorder in remission and understand its practical value.

## 2. Materials and Methods

### 2.1 General Data

A total of 60 patients with bipolar disorder in remission during outpatient and discharge follow-up at Wenzhou Seventh People’s Hospital from December 2020 to March 2022 were enrolled.

Inclusion criteria: (1) 20–65 years old, primary school education or above; (2) with bipolar disorder according to Diagnostic and Statistical Manual of Mental Disorders-5 (DSM-5) diagnostic criteria; (3) maintained clinical remission for more than 2 months; and (4) Young Mania Scale score was less than 7, and the Hamilton Depression Rating Scale-17 (HAMD-17) depression scale score was less than 8.

Exclusion criteria: (1) mental retardation; (2) neurocognitive dysfunction caused by other diseases; (3) a history of drug abuse and/or dependence; and (4) have received Modified Electroconvulsive Therapy (MECT) within 30 days, or are currently receiving Transcranial Magnetic Stimulation (TMS), and have previously received regular psycho-behavioral therapy.

This study was a randomized controlled experiment, and all enrolled subjects were randomly assigned to the study group or the control group. The control group received general drug therapy, and the study group received group psychoeducation combined with drug therapy, with 30 participants in each group. The clinical filing number is MR-33-21-001664 and the clinical filing address is https://www.medicalresearch.org.cn/ in the Chinese National Universal Health Insurance Information Platform - Medical Research Registration and Filing Information System. Study group: 21 males and nine females, aged 20–64 years (45.5 ± 10.1 years), disease course 2–15 years (6.2 ± 3.3 years). Control group: 20 males and 10 females, aged 20–65 years (45.0 ± 10.0 years), disease course 2–14 years (6.0 ± 4.0 years). There were no significant differences in the general data between the two groups of patients before the intervention.

### 2.2 Methods of Intervention 

The subjects were first assessed at baseline. Subjects in the study group were admitted to a psychoeducational group of 6 to 8 people each, receiving five 90-minute sessions once a week. Differences in adherence between the two groups were observed after the intervention.

Intervention content of the study group:

(1) Setting up a group work team, consisting of one psychiatrist, one psychotherapist, and two nurses, who were trained using the group psychoeducation manual.

(2) Conducting group psychoeducation: one time per month, 2–3 hours each time, six times in total. Psychoeducation topics included understanding of bipolar disorder, medication adherence, avoiding substance abuse, recognizing early prodromal symptoms and preventing relapse, establishing daily habits, and stress management. Emotional support and understanding were also provided to patients to make them feel accepted and respected. Patients were encouraged to share their experiences and feelings, to support and encourage each other, and to build a positive group atmosphere.

(3) Organizing group activities: patient skills and strategies to cope with the disease, such as emotional management, stress coping, problem solving, and social skills were taught. Practical exercises were conducted through role-playing and group discussions to improve patients’ practical skills. Group activities were organized once every 2 weeks, 2–3 hours each time, six times in total. Group activities included outdoor sports, artwork, handicrafts, role-playing, etc., aiming to promote interaction and communication among patients and improve teamwork and self-expression.

(4) Individual psychotherapy: individual psychotherapy was conducted once a week for 50–60 minutes each time, for a total of 12 sessions. Individual psychotherapy included cognitive-behavioral therapy, interpersonal psychotherapy, and psychodynamic therapy, etc., aiming to help patients solve their emotional problems, interpersonal problems, and psychological adaptation problems.

(5) Family education: family members were invited to participate in group therapy to provide family support and education. Family members were encouraged to understand the patient’s condition and needs, to master the skills of communicating and getting along with the patient, and to work together to create a good family environment for the patient’s recovery. Family education was conducted once a month, 1–2 hours each time, for a total of six times. The content of family education includes knowledge of BD, family support and communication skills, etc., aiming to help the patient’s family better understand and support the patient.

(6) Regular follow-up: after the treatment, patients in the experimental group were followed up regularly to understand their condition, recovery, and quality of life, and to provide necessary support and guidance.

(7) Assessment and feedback: during the treatment process, the patients’ conditions were regularly assessed, such as changes in Insight and Treatment Attitudes Questionnaire (ITAQ) scale, Morisky medication adherence scale, and Self-rating Depression Scale (SDS) scores. The treatment program was adjusted according to the assessment results, and patients were given timely feedback and encouragement.

In the control group, only drug therapy was used. The main adjuvant drugs were mood stabilizers (valproate, lithium salt), atypical antipsychotics and antidepressants, and benzodiazepines. Subjects were randomly assigned to enrollment according to the randomization schedule and completed baseline and post-interventional assessments. Group work was performed independently by two specially trained social workers and psychotherapists, but they did not participate in any assessment.

### 2.3 Observational Indicators and Evaluation Criteria

Patient treatment adherence score: the Morisky Medication Adherence Scale was used, which was first proposed by Morisky *et al*. in 1986 [11]. It used 1–5 points corresponding to 1–5 levels, with higher scores representing the more desirable degree of patients’ adherence to the instructions of the medical staff. Due to the small number of questions, the Morisky Medication Adherence Scale (MMAS-4) is more suitable for rapid screening, but does not provide in-depth analysis of adherence behavior. The scale has good internal consistency and retest reliability, with a Cronbach’s α coefficient of 0.752.

Assessment of patients’ self-knowledge and treatment attitude: the ITAQ [12] was used, which mainly focuses on patients’ subjective knowledge of the disease and subjective attitude toward treatment, with the total score ranging from 0 to 22, with a higher score representing better self-knowledge and treatment attitudes. The scale has good internal consistency and retest reliability, with a Cronbach’s α coefficient of 0.861.

Medication adherence: this was measured by the Morisky Medication Adherence Scale (MMAS-8), which is an expanded version of the Morisky *et al*. in 2008, based on the original MMAS-4 scale [13]. It consists eight entries and a total score ranging from 0–8, with higher scores representing better adherence. The MMAS-8 is designed to provide a more detailed and accurate assessment of medication adherence, and is particularly suited to clinical studies that require the assessment of multidimensional factors such as patient behavior, psychological factors and environmental influences. The scale had good internal consistency and retest reliability, with a Cronbach’s α coefficient of 0.741.

Patients’ overall treatment: the Sheehan Disability Scale (SDS) [14] was used to assess the overall treatment of the patients, in which SDS1 mainly assessed the impact of clinical symptoms on the ability to work, SDS2 mainly assessed the impact of clinical symptoms on social life, and SDS3 mainly assessed the impact of clinical symptoms on family life, with the maximum scores of 10 and 30 points. Higher scores represent more severe disability. The scale had good internal consistency and retest reliability, with a Cronbach’s α coefficient of 0.910.

Disease intervention coping styles: the Trait Coping Styles Questionnaire (TCSQ) [15] was used for assessment, which is mainly divided into two categories, positive coping and negative coping. The scale had good internal consistency and retest reliability, with a Cronbach’s α coefficient of 0.709.

### 2.4 Statistical Analysis 

SPSS 20.0 (IBM Corp., Armonk, NY, USA) was used for statistical analysis. Count data were expressed in frequency, and differences between groups were analyzed using Fisher’s exact method (for small sample data). The continuous data were described by Shapiro-Wilk normality test, and those who met the normal distribution were described as mean ± standard deviation (Mean ± SD), and the independent samples *t*-test was used to compare the differences between groups. Non-normal data are presented as medians (interquartile ranges, IQR) and are compared between groups by Mann-Whitney U test. The threshold for statistical significance is defined as *p* < 0.05.

## 3. Results

### 3.1 Comparative Analysis of Treatment Compliance 

The Table 1 shows a comparison of treatment adherence between the control and study groups. There was no statistically significant difference in pre-intervention scores between the two groups (*p* > 0.05). However, the change in post-intervention adherence score (ΔPost-Pre) showed a significant difference between the two groups (*p* < 0.05). These results suggest that the intervention had a significant positive effect on treatment adherence in the study group compared to the control group.

**Table 1.** **Comparison of treatment compliance**.

Group	Pre-intervention	ΔPost-Pre
Control group	2.20 (2.00–2.60)	0.80 (0.50–1.20)
Study group	2.10 (1.85–2.40)	2.30 (2.10–2.60)
U	400.5	142.5
*p*	0.921	<0.001

### 3.2 Comparative Analysis of ITAQ Scores and Drug Compliance 

Table 2 shows a comparison of the ITAQ and Morisky scales for medication adherence scores between the control and study groups. For ITAQ score and Morisky, the difference in pre-intervention scores was not statistically significant (*p* > 0.05), however, the change in post-intervention adherence score (ΔPost-Pre) showed a significant difference between the two groups (*p* < 0.05).

**Table 2.** **Comparison of ITAQ and Morisky scale of drug compliance scores**.

ITAQ score				
	Control group (n = 30)	Study group (n = 30)	U	*p*
Pre-intervention	10.0 (8.0–12.0)	12.0 (8.0–14.0)	300.0	0.12
ΔPost-Pre	3.0 (0.0–6.0)	8.0 (6.0–10.0)	268.0	0.02
Morisky score				
	Control group (n = 30)	Study group (n = 30)	U	*p*
Pre-intervention	3.0 (3.0–5.0)	3.0 (2.5–4.0)	351.5	0.15
ΔPost-Pre	2.0 (1.0–3.0)	4.0 (3.0–5.0)	221.0	0.002

ITAQ, Insight and Treatment Attitudes Questionnaire.

### 3.3 Comparison of Total Sheehan Disability Scale Scores and Sub-Scores After Intervention

After intervention, the total SDS1, SDS2, SDS3, and SDS scores in the study group were 8.5 ± 1.2, 8.0 ± 1.5, 7.9 ± 2.0, and 25.5 ± 4.3, respectively, which were significantly higher than those in the control group (3.2 ± 0.7, 3.0 ± 0.8, 2.8 ± 1.2, 15.4 ± 3.4, all *p* < 0.001). See Table 3.

**Table 3.** **Comparison of total SDS score and each sub-item score after intervention**.

Group	SDS1	SDS2	SDS3	SDS total score
Study group	8.5 ± 1.2	8.0 ± 1.5	7.9 ± 2.0	25.5 ± 4.3
Control group	3.2 ± 0.7	3.0 ± 0.8	2.8 ± 1.2	15.4 ± 3.4
*p*	<0.001	<0.001	<0.001	<0.001

SDS, Self-rating Depression Scale.

### 3.4 Statistical Comparison of the Proportion of Coping Styles After Intervention

The proportion of positive coping styles in study group was significantly higher than that in the control group (93.3% *vs*. 50.0%, *p* =0.001). See Table 4.

**Table 4.** **Statistical comparison of coping style proportion between the two groups after intervention**.

Group	Positive coping	Negative coping
Study group	28	2
Control group	15	15
*p*	<0.001	

## 4. Discussion

Bipolar disorder is the most common type of mood disorder. Current treatment methods are not ideal and relapse is common, which may be related to patients’ low adherence to medication and irregular medication [16]. Therefore, improving the treatment compliance of bipolar disorder patients, enhancing self-management ability, improving the social support system, and promoting the patients’ mental health adaptation are the keys to optimize the treatment effect of bipolar disorder. The group psychoeducation-based intervention model adopted in this study plays an important role in the continuation of treatment for bipolar disorder patients in remission.

The intervention based on the group psychoeducation model in the study group, including recognition of bipolar disorder, drug compliance, avoidance of substance abuse, identification of early prodromal symptoms and prevention of relapse, and establishment of daily habits and stress management, was an enrichment and expansion of the previous psychoeducation model, and also more structured and targeted. Therefore, the group psychoeducation model is used as a health education manual specifically developed for patients with bipolar disorder in remission. According to the comparison of treatment compliance, treatment compliance in the study group was higher than in controls after intervention. Casellas *et al*. [17] also indicated in their study that group psychoeducation helps to enhance patients’ treatment adherence, similar to the results of this study. The reasons for this were that the group atmosphere enhanced patients’ sense of belonging and social support, reduced resistance to treatment, and the group interaction helped patients to monitor and encourage each other, which improved overall treatment adherence.

At the same time, patients’ ITAQ and medication compliance (Morisky scale) scores were compared, and it was found that the self-awareness, treatment attitude, and medication compliance of the research group were higher than those of the control group after the intervention. This indicates that continuous intervention using the group psychoeducation model for patients with bipolar disorder in the remission stage can significantly improve treatment compliance, improve insight and treatment attitude, and ensure drug compliance. The results of this study are further supported by the study of Zyto *et al*. [18], which indicated that group psychoeducation was effective in enhancing patients’ attitudes toward treatment and self-management, and by the study of Okazaki *et al*. [19], which indicated that attitudes toward medication and program satisfaction gained through psychoeducation affected long-term medication adherence.

In addition, the overall patient treatment i.e., SDS total score and scores of each entry were also compared between the two groups. After intervention, the total SDS1, SDS2, SDS3, and SDS scores in study group were higher than those in the control group. It is suggested that continuous intervention with the group psychoeducation model is of certain value in reducing the score of the whole disability scale and improving the patient prognosis in the remission stage. A study by Morriss *et al*. [20] indicated that psychoeducation and instruction in practicing illness management skills in a family or group setting was more effective in reducing relapse rates and alleviating symptoms of mental illness than implementing the same strategies individually, similar to the results of this study. The reasons for this are that group education enhances patients’ knowledge of their dysfunction and self-management ability, group support helps patients better cope with the effects of dysfunction, and the integrated treatment program meets the physical, psychological, and functional needs of patients.

Finally, the proportion of positive coping styles in the study group after intervention was significantly higher than that in the control group. It was further demonstrated that continuous group psychoeducation intervention for patients with bipolar disorder in remission can fundamentally change their subjective attitude towards the disease, and thus has value for improving treatment compliance and ensuring clinical treatment effect. Maçkalı *et al*. [21], in their study, indicated that group therapy intervention had a positive impact on participants’ self-awareness, self-acceptance, and self-perception, further suggesting that group therapy intervention corrected misperceptions and inappropriate coping styles, and changed patients’ subjective attitudes toward their illnesses, which is similar to the results of the present study. In conclusion, the core strength of group psychoeducation lies in the creation of a supportive and interactive therapeutic environment, which helps to improve patients’ cognition and behavior, thus bringing about positive effects in many aspects mentioned above. This provides an effective method for the continuation of treatment for bipolar disorder patients in remission.

This study investigated the impact of PM-based group work on the treatment compliance of patient in remission, which facilitates rehabilitation [22] and increases the efficacy of drugs, and has important implications for the clinical diagnosis and treatment of bipolar disorder [23]. It is better to introduce relevant knowledge of the disease to bipolar patients through group scenario intervention [24], share learning experiences, identify patients’ signals of undesirable mood fluctuations in a timely manner [25], and teach patients and their families simple disease self-management methods [26], which can reduce the impact of acute stress events [27] and reduce stigma [28]. This study will promote the practical clinical application of group work based on the group psychoeducation model in the psychiatry department. It has definite clinical effect, which can enhance the curative effect of drugs, alleviate the pain of patients with bipolar disorder, and lighten the burden of families, and has obvious positive effects on social development.

Although the present study has achieved certain results, there are some limitations. First, the sample size of this study is relatively limited, and the patients with bipolar disorder in remission were from a specific region, so the extrapolation and representativeness of the results may be affected to some extent. Second, the study only focused on the short-term effects of the intervention and lacked a long-term follow-up assessment to determine the durability of the intervention effects. In addition, this study only used subjective assessment tools such as self-assessment scales and lacked the use of more objective assessment tools such as physiological indicators and cognitive function tests, which may have affected the accuracy of the results. Finally, this study failed to comprehensively consider the influence of patients’ social support system, economic status, and other factors on the effect of intervention.

Future studies can be improved and expanded in the following aspects. First, expanding the sample size and recruiting subjects from different regions and backgrounds to improve the generalizability of the results. Second, extending the follow-up period to assess the durability of the long-term intervention effects. Third, we will combine physiological and neuropsychological tests and adopt various evaluation methods to evaluate the intervention effect more comprehensively and objectively. Fourth, we will explore the application of different types of group work models in bipolar disorder patients and conduct comparative studies with individual models. Fifth, we will carry out qualitative research to analyze the mechanism of group psychoeducation and group work models in depth. Further research and practical exploration are expected to provide more effective treatment programs for the long-term management of bipolar disorder patients.

## 5. Conclusion

In summary, group psychoeducation model continuous intervention for patients with bipolar disorder in remission can significantly improve treatment compliance, improve insight and treatment attitudes, ensure drug compliance, and reduce the degree of mental disability.

## Data Availability

The data and materials of this experiment are available from the corresponding author on reasonable request.
